# Optimization of protein extraction and two-dimensional gel electrophoresis profiles for the identification of *Cordyceps sinensis* and other similar species

**DOI:** 10.1371/journal.pone.0202779

**Published:** 2018-08-22

**Authors:** Roy Chi Ho Chan, Steven Sai Wo Lam, Fiona Long Yan Fong, Dominic Tak Wah Chan, Fred Wang Fat Lee, Eric Tung Po Sze

**Affiliations:** 1 School of Science and Technology, The Open University of Hong Kong, Hong Kong Special Administrative Region, the People’s Republic of China; 2 Department of Chemistry, The Chinese University of Hong Kong, Hong Kong Special Administrative Region, the People’s Republic of China; Shantou University Medical College, CHINA

## Abstract

Given that Chinese materia medica (CMM) is expensive and rare, people take tremendous risk to adulterate and falsify *Cordyceps sinensis* with counterfeit species with similar morphological features. It is thus essential to develop new methods to identify the authenticity of *Cordyceps sinensis*. It is hypothesized in this study that *Cordyceps sinensis* possesses certain protein biomarkers distinct from its counterfeits, which can be identified by proteomic technologies for authentication purposes. This is the first study that aims to optimize the conditions for extracting proteins from *Cordyceps sinensis*, a hybrid of fungal-animal CMM, and to compare the two-dimensional gel electrophoresis (2-DE) profiles between different *Cordyceps* species. Two different protein extraction buffer systems, namely, phenol/sodium dodecyl sulfate (SDS) buffer or lysis buffer, were evaluated, where the preparation using lysis buffer yielded better protein content. The results also showed that extraction with lysis buffer without pre- or post-washing of samples was the most effective protocol, with over 220% of protein yield and 819 protein spots detected on a 2-DE gel. Moreover, the results demonstrated that *Cordyceps sinensis* possesses protein biomarkers distinct from its counterfeits, and these biomarkers are not source- or origin-dependent, strongly supporting the feasibility of using identified biomarkers as indicators for authentication of *Cordyceps* species. The findings of this study warrant further investigations on the structural identification of protein biomarkers of *Cordyceps* species.

## Introduction

*Cordyceps sinensis* is also known as “Dong Chong Xia Chao”. While it has been deemed as a cornerstone of Chinese material medica (CMM) for centuries, people from Western countries have only come to know about *Cordyceps sinensis* since the 1990s. *Cordyceps sinensis* is a composite CMM consisting of a stromata of fungus parasitized on subterranean caterpillar or the fruiting bodies of truffles [1]. It has been explicitly demonstrated to have various beneficial health effects, including for the treatment of renal dysfunction, relieving hyperglycemia, ameliorating hyperlipidemia and strengthening immunity [2–8].

*Cordyceps sinensis* is rare and exotic, as it can barely be found at the high altitudes in the Qinghai-Tibetan plateau [1,2]. Regardless of the restricted geographical distribution and specific habitat of *Cordyceps sinensis*, the increasing demand, together with devastating human factors and global pollution, has diminished the yields, resulting in exorbitant selling prices. This monetary incentive has instigated people to adulterate and falsify *Cordyceps sinensis* with counterfeits with similar morphological features [9], which may consequently dampen the quality and safety of CMM products.

Being an expensive and rare CMM, people currently take tremendous risk to adulterate and falsify *Cordyceps sinensis* with counterfeit species with similar morphological features for sale [9] in pharmacies, due to their lower price or feasibility to be cultivated [10]. Not only has different efficacy of counterfeit *Cordyceps sinensis* been observed, but detrimental health effects have also been observed in certain counterfeit species [3, 11–13].

To authenticate *Cordyceps sinensis*, some researchers have determined the chemical components of samples with various analytical methods such as high-performance liquid chromatography [14,15], Fourier transform near-infrared (FT-NIR) spectroscopy [16] and tandem mass spectrometry [15,17]. However, these physiochemical identification and chromatographic techniques generally require great consumption of samples, which are thus costly and impractical for medicinal retailers and traders to apply in routine quality control processes. Additionally, chemical markers of *Cordyceps sinensis* for analysis are lacking, which hinders the development of these chemical techniques [18]. In this context, microscopic examination can be adopted to differentiate *Cordyceps sinensis* from its counterfeits [19,20]. Although microscopy requires fewer samples, it is an ambiguous technique that depends extensively on the knowledge and experience of specialists. Molecular [21] techniques such as DNA identification may be used for the authentication of *Cordyceps* species, but these methods are affected by the factors of doping.

To address this problem, it is necessary to develop a new testing method that can offer a cost-effective and user-friendly approach for the authentication of *Cordyceps sinensis*. It is hypothesized that *Cordyceps sinensis* possesses certain protein biomarkers distinct from its counterfeits, which can be identified by proteomic technologies for authentication purposes. The key to success in proteomic analysis is greatly determined by the step of protein extraction [22]. While protocols for protein extraction from various animal and plant tissues are available in the literature [23–25], a protocol specifically for *Cordyceps sinensis* is not well defined. This study thus aims to develop an optimized protocol for extracting proteins specifically from *Cordyceps sinensis*. Two-dimensional gel electrophoresis (2-DE) profiles of *Cordyceps sinensis* and its counterfeit species, as well as *Cordyceps sinensis* from different sources, were compared in this study. To the best of our knowledge, this is the first comparative study to optimize a protein extraction method for *Cordyceps sinensis* and to evaluate the 2-DE profiles of *Cordyceps* species.

## Materials and methods

### *Cordyceps sinensis* and other *similar* species

**Fig 1:**
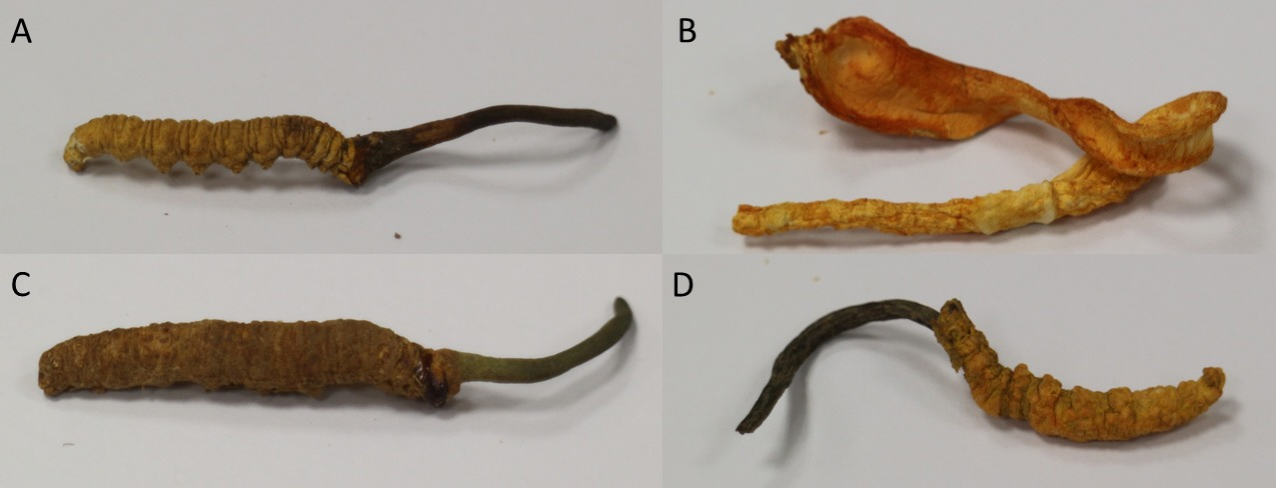
Photo of *Cordyceps* species. (A) *Cordyceps sinensis*; (B) *Cordyceps militaris;* (C) *Cordyceps hawkesii*; (D) *Metacordyceps taii*.

We have conducted a pilot survey with some retail chain stores and market surveillance of other small retailers. *Cordyceps* species including *Cordyceps militaris*, *Cordyceps hawkesii* and *Metacordyceps taii* are the major species with similar morphological features (Fig 1) that have been used for counterfeiting in local markets and were chosen for this study. All *Cordyceps* species with similar morphological features were obtained from local retail markets and/or donors, with the details of their purchase located in Table 1. All samples were stored in controlled temperature and humidity at 20 ± 3°C and below 20%, respectively. Before extraction, the sample was ground to a fine powder with a mortar and pestle in liquid nitrogen prior to extraction [26], and methanol was used to clean the mortar and pestle after each run to avoid cross contamination. Each sample of *Cordyceps* species was identified genetically (Table 2). The similarities of all tested *Cordyceps* species were greater than 97% when compared with the sequence in NCBI GenBank.

**Table 1:** Details of the purchased *Cordyceps* species.

Sample	Species	Purchase locations	Source origin
1	*Cordyceps sinensis*	Imperial Bird’s Nest International Co. Limited	Tibetan Plateau
2	*Cordyceps sinensis*	Hing Kee Java Edible Bird's Nest Company Limited	Tibetan Plateau
3	*Cordyceps militaris*	Imperial Bird’s Nest International Co. Limited	Unknown
4	*Cordyceps hawkesii*	Imperial Bird’s Nest International Co. Limited	Unknown
5	*Metacordyceps taii*	Wanfeng Chinese and Western Medicine	Unknown

**Table 2:** Identification of *Cordyceps* species by DNA sequencing.

Species	NCBI GenBank Accession No.	Length (bp) of ITS1 and ITS4 region	Similarity (%)
*Cordyceps sinensis*	EU570928.1	579	99.1%
*Cordyceps militaris*	AY245634.1	567	99.1%
*Cordyceps hawkesii*	AJ536571.1	632	99.8%
*Metacordyceps taii*	EF495101.1	533	97.9%

### Protein extraction and quantitation

An overview of the protein extraction method for all *Cordyceps* species by the use of various protocols is presented below. Each protocol was repeated seven times for the study of protein yield by mass.

Phenol/sodium dodecyl sulfate (SDS) buffer (Protocol A) extraction was established based on the protocol described by Ishtiaq et al. [25]. The ground sample was re-suspended in phenol and SDS buffer (30% sucrose, 2% SDS, 0.1 M Tris-HCl, pH 8.0 and 5% 2-mercaptoethanol) (1:1 by volume). The phenol phase was collected after centrifugation at 10,000 rpm for 10 minutes at room temperature, and the phenol extraction was repeated twice. Then, 0.1 M ammonium acetate in methanol was added to the phenol phase (5:1 by volume), and the mixture was kept at -20°C. Precipitated proteins were recovered by centrifugation at 10,000 rpm for 10 min at 4°C. The pellet was washed with 0.1 M ammonium acetate in methanol and 80% acetone in water twice. The pellet was re-suspended in buffer containing 7 M urea, 2 M (w/v) thiourea, 2% 3-[(3-cholamidopropyl)dimethylammonio]-1-propanesulfonate (CHAPS), 1% (v/v) 3/10 Ampholyte (Bio-Rad, California, USA), 40 mM Tris and 10 mM Acrylamide. The protein content was then quantified.

Lysis buffer (Protocol B) extraction was established based on the protocol described by Jiang et al. [27], where lysis buffer (7 M urea, 2 M thiourea, 4% (w/v) CHAPS, 40 mM dithiothreitol (DTT), 0.2% (w/v) ampholytes (pH 3–10) and 40 mM Tris base) was added to the sample and sonicated on ice for 10 minutes. The extracted mixture was centrifuged at 15,000 x g for 5 minutes at room temperature. The protein content in the supernatant was measured.

TCA/acetone pre-washing followed by lysis buffer extraction (Protocol C) was developed by the procedure described by Sun et al. [28], where the sample was pre-washed with 20% trichloroacetic acid (TCA) in acetone (1:5 by volume) for 12 hours at -20°C. The mixture was then centrifuged at 15,000 x g for 15 minutes at room temperature. The pellet was re-suspended in acetone with 0.2% DTT and incubated for 15 minutes at -20°C and then centrifuged at 15,000 × g for 15 minutes at room temperature. The above washing step was repeated twice. The final pellet was re-suspended in lysis buffer, and protein extraction using protocol B was performed.

Lysis buffer extraction followed by TCA/acetone post-washing (Protocol D) was modified by the protocol described by Wang et al. [29], where protein was first extracted by following Protocol B. The resulting supernatant was mixed with 20% TCA in acetone (1:5 by volume) and incubated at -20°C overnight. The mixture was centrifuged at 15,000 xg for 30 minutes at room temperature, and then the pellet was re-suspended in acetone with 0.2% DTT and incubated at -20°C for 30 minutes. The suspension was centrifuged at 15,000 x g for 30 minutes at room temperature. The washing step was repeated twice. The pellet was re-suspended in lysis buffer.

Protein concentration of the extract obtained by Protocols A-D above was quantified as described in the study of the Bradford assay [30].

### Two-dimensional electrophoresis

Immobilized pH gradient (IPG) strips (17 cm length, 0.5 mm thickness) with a linear gradient from pH 4–7 (Bio-Rad) were rehydrated with protein extracts containing 500 μg of protein for 15 hours. Isoelectric focusing (IEF) was carried out at 20°C using a Bio-Rad PROTEAN i12^TM^ IEF cell system, with the voltage program as follows: 500 V for 17 h, 1,000 V for 1 h, 10,000 V for 3 h, and 10,000 V up to a total of 43,000 V/h. After IEF, the IPG strips were equilibrated in a buffer solution composed of 6 M urea, 2% SDS, 0.375 M Tris-HCl (pH 8.8), 20% glycerol and 2% (w/v) DTT, followed by a solution of 6 M urea, 2% SDS, 0.375 M Tris-HCl (pH 8.8), 20% glycerol and 2.5% iodoacetamide for 15 minutes. The second-dimension SDS-polyacrylamide electrophoresis was carried out using a Bio-Rad PROTEAN^®^ II xi cell at 400 V for 3 hours. The gel was fixed in fixation solution containing 10% (v/v) acetic acid and 40% (v/v) methanol for silver staining.

### Gel image analysis

The resulting 2-DE gels were scanned with a high-resolution scanner (Bio-Rad ChemiDoc™ MP) and analyzed by Melanie 2-D electrophoresis analysis software (Bio-Rad). Smoothness, saliency and the minimum area of protein spots detected were adjusted for optimal clarity. Spots detected near the edges of the gels were deleted manually to avoid erroneous interpretation. Each of the identified spots was marked and numbered.

### Statistical analysis

Matches were analyzed with the analysis of variance (ANOVA) test. Tukey’s multiple test was also used for pairwise comparison, with a significance level of p < 0.01 being chosen as significant. Statistical calculations were performed using GraphPad Software Prism Version 6.04 (San Diego, CA, USA).

## Results and discussion

### Protein extraction efficiency of *Cordyceps sinensis* using different extraction buffer systems

The critical and determining step for the success of proteomic analysis is commonly deemed to be the protein extraction step, in which the choice of extraction buffer is of importance to the yield of the proteins extracted. Previous studies have overwhelmingly reported the optimal conditions for protein extraction for animal tissues [31], but information specifically for *Cordyceps* species, a hybrid of fungal-animal CMM, is lacking. By making reference to the literature about some recalcitrant plant tissues, phenol/SDS [24,25] and lysis [27,32] buffer systems were chosen for this study.

The results of proteins extracted from *Cordyceps sinensis* using Phenol/SDS extraction buffer (Protocol A) and lysis buffer (Protocol B) were found to be 7.0% and 22.7% by mass, respectively, where 220% more proteins were extracted by Protocol B. This may be caused by urea and thiourea in the lysis buffer having a higher efficiency in breaking the hydrogen bonds and hydrophobic interactions between and within proteins contained in *Cordyceps sinensis* than the use of sucrose in the SDS buffer. Some research studies have shown that phenol results in poor protein solubilization [33]. Moreover, ammonium acetate in methanol for use in phenol/SDS buffer would not only remove non-protein contaminants such as nucleic acids but also cause a substantial loss of proteins [34–36]. Based on our results, the phenol method was not suitable for protein extraction from *Cordyceps sinensis*. Although protein degradation contained in the samples would be highly variable and may depend on factors such as the conservation method, the yields of protein extracted by lysis buffer in this study was found comparable with another study’s result [32].

### Effects of pre-washing or post-washing for protein extraction with lysis buffer

**Fig 2:**
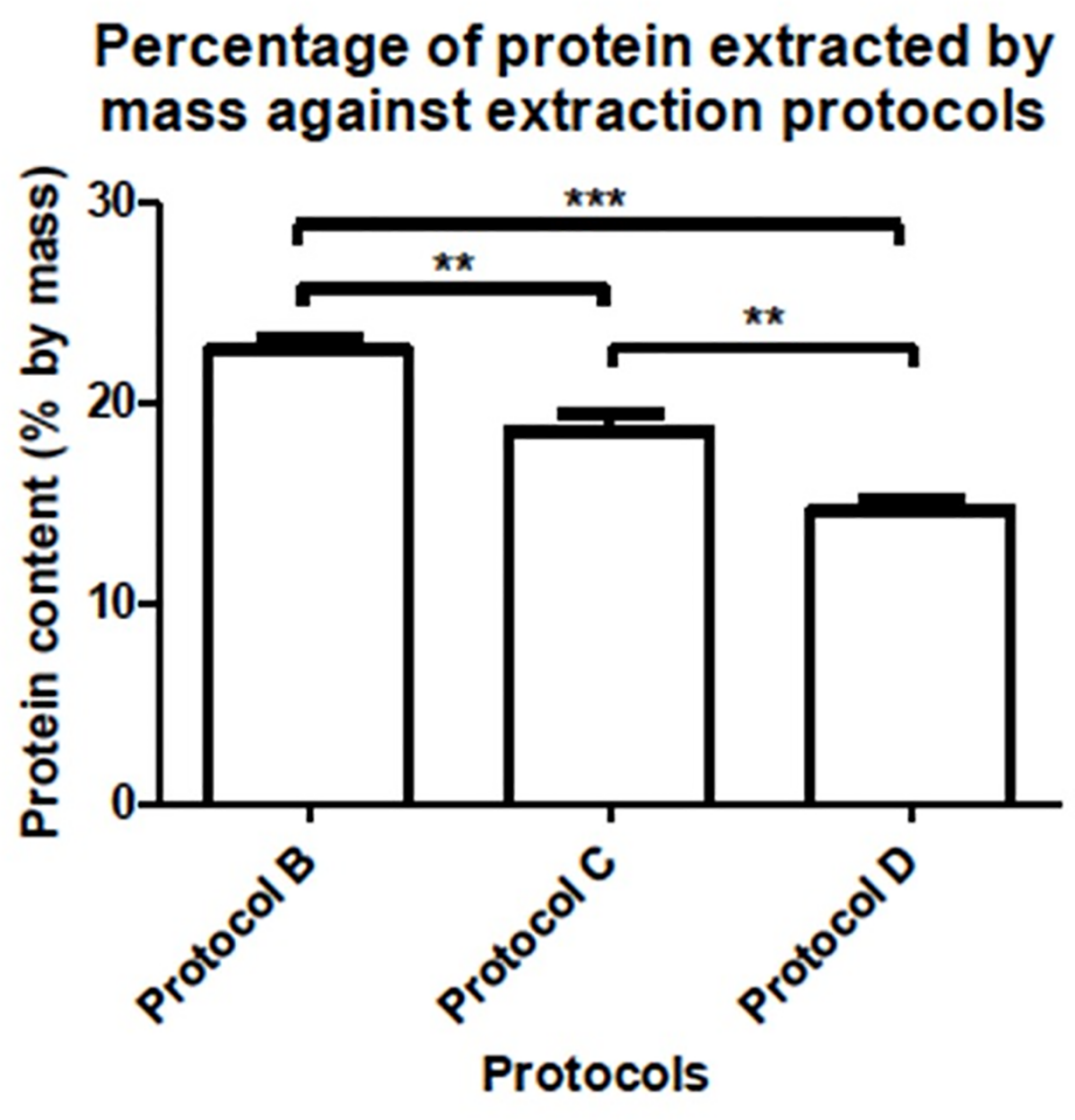
Statistical comparison test for the significant differences in extracted protein content (% by mass) using different lysis buffer extraction protocols. The Tukey’s test was carried out to compare the means of extracted protein in different protocols. The results are presented as the mean ± standard deviation. Error bars indicated standard deviation (*n* = 7). **p < 0.01 and ***p < 0.001 in comparison between the different protocols.

**Fig 3:**
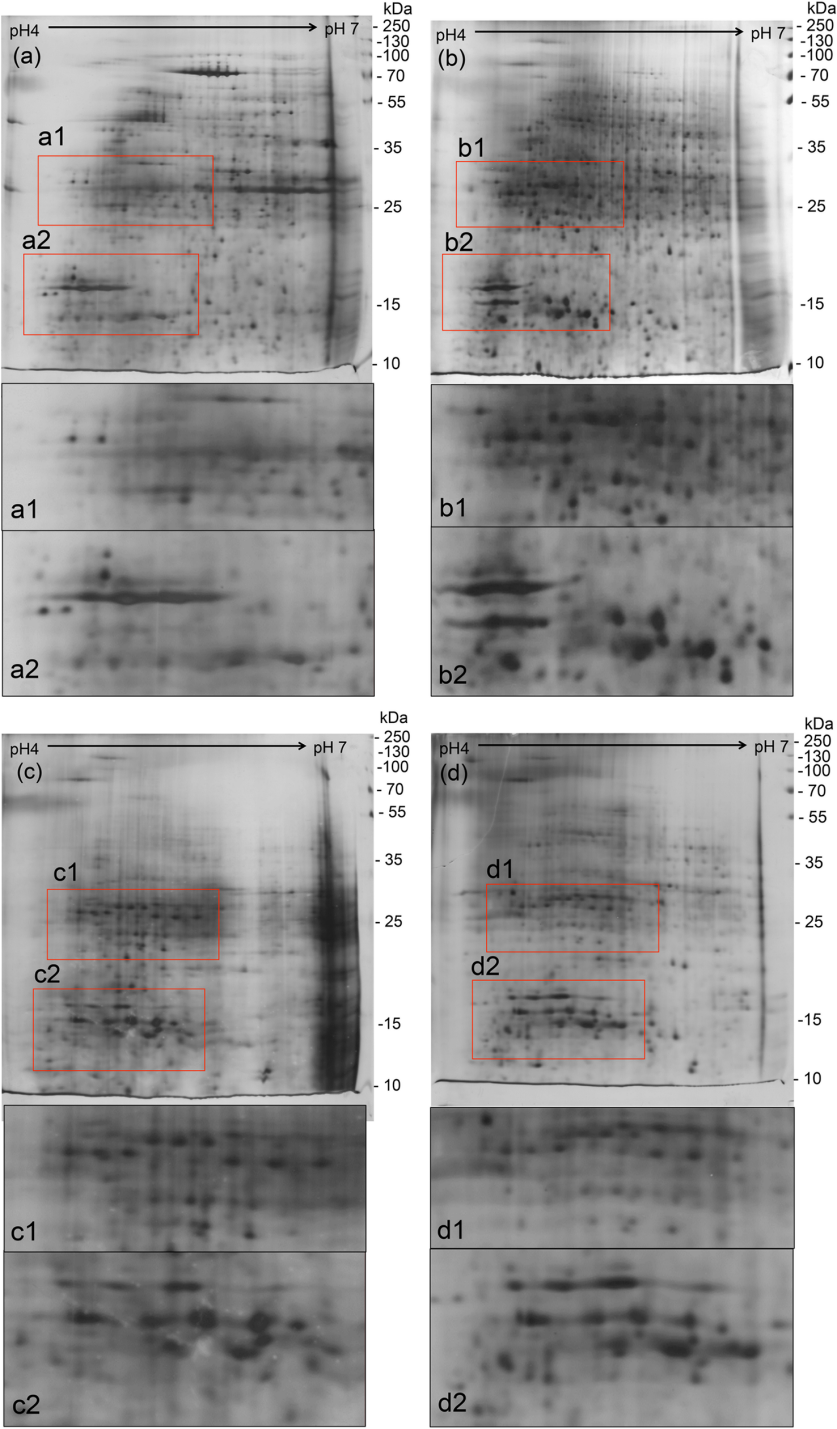
2-DE gel profiles of *Cordyceps sinensis* using different extraction protocols. (a) Protocol A, (b) Protocol B, (c) Protocol C, and (d) Protocol D.

We then further investigated whether pre-washing or post-washing of samples with TCA/acetone would enhance the extraction of proteins in *Cordyceps sinensis* with lysis buffer. The pre-washing and post-washing steps were intended to minimize interferences in the samples and could thus improve the quality of the 2-DE profiles, which is broadly employed for the cleanup of samples and precipitating proteins in the literature [28, 37, 38]. From the results obtained (Fig 2), however, both pre-washing and post-washing cleanup processes were shown to have substantial protein loss (18% and 35% mass loss, respectively, when compared with direct lysis buffer extraction without cleanup). Statistical test results (with a significance level of p < 0.01) also supported a significant difference in the protein content extracted. The 2-DE profiles (Fig 3) also demonstrated a severe reduction in high molecular weight protein spots by the cleanup processes, but with no improvement to the resolution and clarity of the resulting gel images. We suspected that some degradation or modification of high molecular weight proteins at low pH was occurring in TCA/acetone [23].

### Comparison of 2-DE profiles of *Cordyceps species*

**Fig 4:**
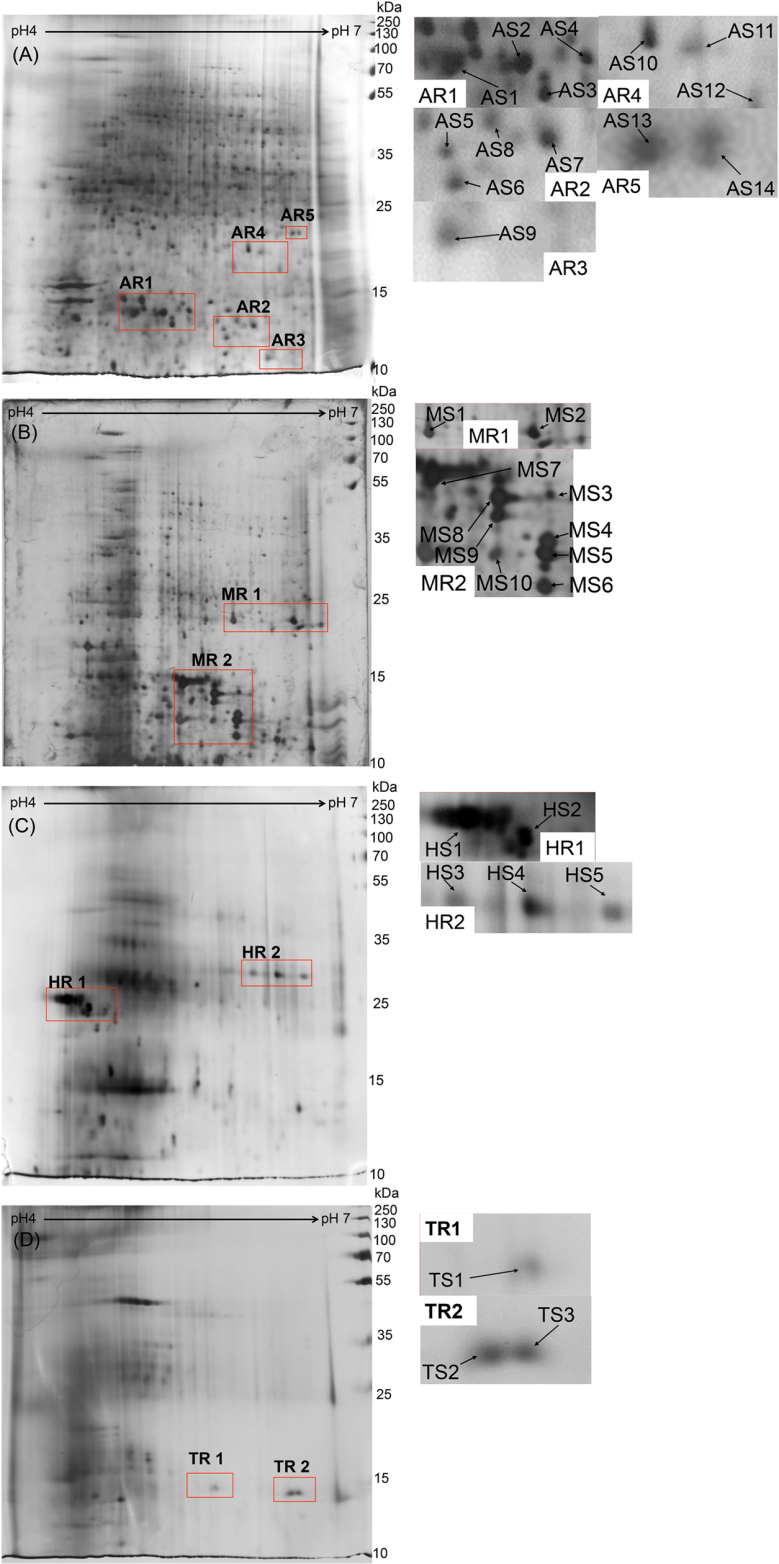
**2-DE gel profiles of (A) *Cordyceps sinensis*, (B) *Cordyceps militaris*, (C) *Cordyceps hawkesii* and (D) *Metacordyceps taii*, with extraction using lysis buffer without any cleanup process.** All gel images were analyzed by Melanie 2-D electrophoresis analysis software.

Proteins were extracted from *Cordyceps sinensis* and other similar species, namely, *Cordyceps militaris*, *Cordyceps hawkesii* and *Metacordyceps taii* using Protocol B. The extracted proteins were separated by 2-DE with gels being stained (Fig 4), and the yield being monitored by a Bradford protein assay. The total number of protein spots was counted, and the 2-DE profiles were compared. The 2-DE profiles obtained from the extracted protein of the four *Cordyceps* species were matched using Melanie 2-D electrophoresis analysis software (Bio-Rad). The protein content of other species (*Cordyceps militaris* 17.2%; *Cordyceps hawkesii* 14.4%; *Metacordyceps taii* 15.4%) and the number of protein spots (*Cordyceps militaris* 701; *Cordyceps hawkesii* 121; *Metacordyceps taii* 128) on the 2-DE gel were significantly different from those of *Cordyceps sinensis* (protein content 22.7%; number of protein spots on gel: 819) (Table 3). Based on the repeat analysis of the 2-DE patterns obtained, *Cordyceps* species showed that the protein spot patterns were greatly different between each other, and we were able to distinguish *Cordyceps sinensis* from other species. To increase the selectivity of the identification, some characteristic regions for each *Cordyceps* species were highlighted as protein marker candidate regions by considering their intensity and location uniqueness.

**Table 3:** Comparison of protein content (% by mass) and total number of protein spots of 2-DE gel images between *Cordyceps sinensis* and other similar species.

Species	Protein content (% by mass)	Number of protein spots
*Cordyceps sinensis*	22.7 ± 1.6	819
*Cordyceps militaris*	17.2 ± 2.6**	701
*Cordyceps hawkesii*	14.4 ± 2.8***	121
*Metacordyceps taii*	15.4 ± 1.1***	128

The gel images were analyzed by Melanie 2-D electrophoresis analysis software. Total number of protein spots was counted where spots detected near the edges of the gels were deleted manually. The results are presented as the mean ± standard deviation.

**p < 0.01 and

***p < 0.001 in comparison with *Cordyceps sinensis*.

As shown in Fig 4, 5 regions (AR1-AR5) from the 2-DE of *Cordyceps sinensis* with 14 specific spots (AS1—AS14); 2 regions (MR1-MR2) from the 2-DE of *Cordyceps militaris* with 10 specific spots (MS1—MS14); 2 regions (HR1-HR2) in *Cordyceps hawkesii* with 5 specific spots (HS1 –HS5); and 3 regions in *Metacordceps taii* (TR1-TR3) with 3 specific spots (TS1 –TS3) were identified as candidate marker regions and candidate markers.

### Verification of candidate protein marker spots by *Cordyceps sinensis* from independent sources

**Fig 5:**
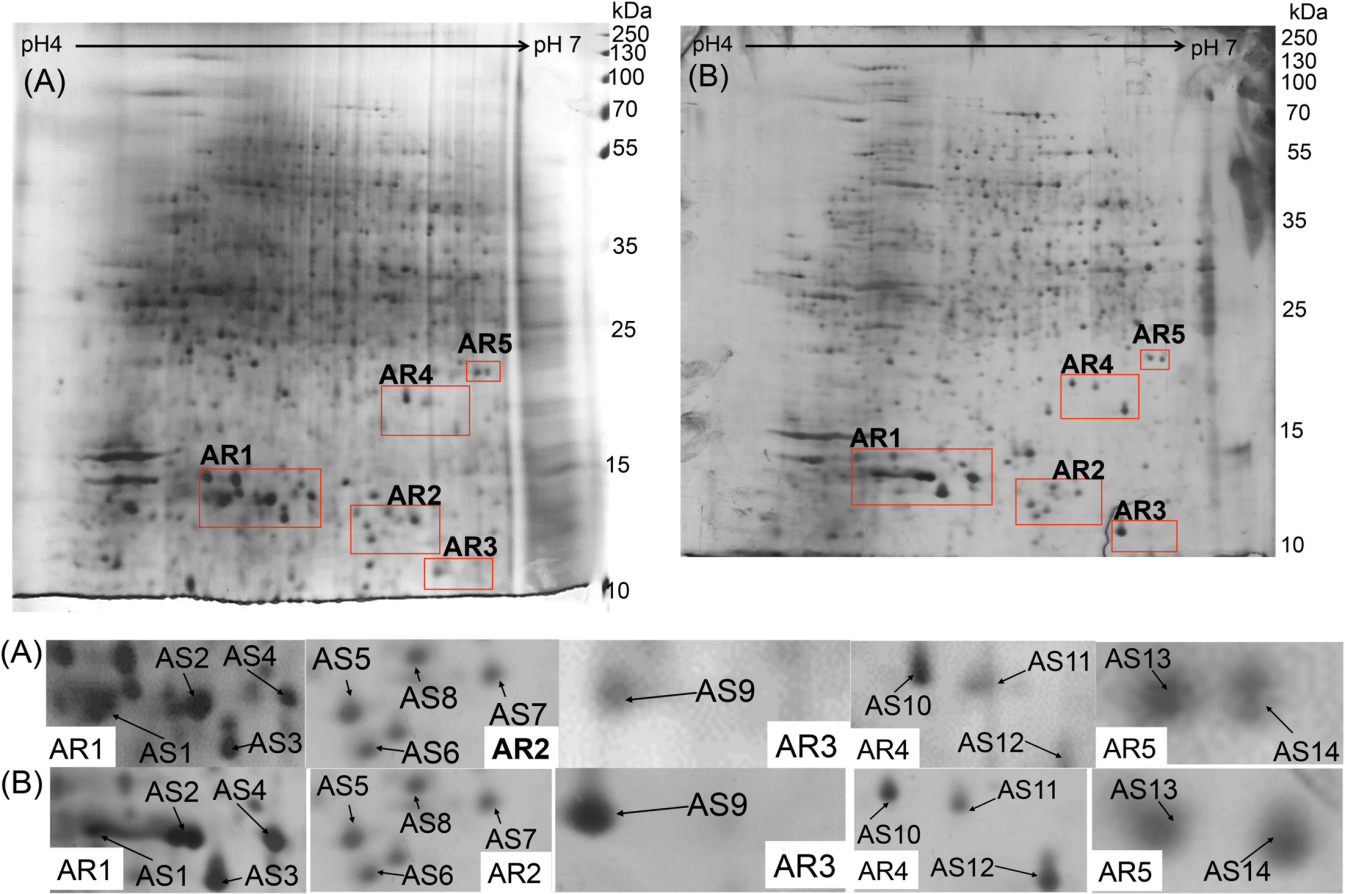
**Comparison of *Cordyceps sinensis* 2-DE profiles of (A) original source and (B) independent source.** All gel images were analyzed by Melanie 2-D electrophoresis analysis software. All candidate protein marker spots (AS1 –AS14) in characteristic regions (RA1—RA5) were found reproducible.

To verify the reproducibility of the protein marker regions, we obtained *Cordyceps sinensis* samples from an independent source and the 2-DE profile was used for comparison with the original sample. As shown in Fig 5, the profile patterns of both 2-DE images generally matched and all candidate marker spots in the selected regions were found to match with the original 2-DE. This suggests that the unique protein biomarkers of *Cordyceps sinensis* are not source- or origin-oriented, strongly supporting the feasibility and practicality of using protein biomarkers for authentication of *Cordyceps sinensis* from other similar species.

## Conclusions

This is the first comparative study to optimize a protein extraction method for *Cordyceps sinensis* and to evaluate the 2-DE profiles of various *Cordyceps* species. We tried using phenol/SDS and lysis buffer extraction systems, where extraction using lysis buffer system was found to have a better protein extraction yield (220% more protein by mass). To optimize the extraction yield and quality of the resulting 2-DE profile of lysis buffer extraction, we studied the effect of TCA/acetone pre-washing and post-washing of *Cordyceps sinensis* sample in conjunction with lysis buffer extraction. Among the three protocols, lysis buffer extraction without any pre-washing and post-washing of the sample was found to be the most effective method in extracting proteins from *Cordyceps* species, with no significant difference in 2-DE profile quality.

Upon optimizing the extraction protocol, we successfully compared the 2-DE profiles of four *Cordyceps* species obtained in the market, and *Cordyceps sinensis* possesses certain candidate protein marker spots distinct from other similar species. These candidate protein marker spots were proven to be reproducible from an independent source, demonstrating the feasibility of using protein markers as indicators for authentication of *Cordyceps sinensis*. Our results should aid future proteomic studies of *Cordyceps sinensis* and other similar species on the identification of biomarkers and their corresponding structural information. Findings of this study provide evidence-based insight and warrant further investigation of the potential biomarkers of the Cordyceps species. The identified biomarkers would be further explored if a fast-screening method could be developed such as screening kits related to color change or antigen-antibody reaction.
